# Prevalence of and factors associated with unplanned pregnancy among women in Koshu, Japan: cross-sectional evidence from Project Koshu, 2011–2016

**DOI:** 10.1186/s12884-020-03088-3

**Published:** 2020-07-09

**Authors:** Son Trung Huynh, Hiroshi Yokomichi, Yuka Akiyama, Reiji Kojima, Sayaka Horiuchi, Tadao Ooka, Ryoji Shinohara, Zentaro Yamagata

**Affiliations:** 1 Department of Health Sciences, School of Medicine, University of Yamanashi, 1110 Shimokato, Chuo, Yamanashi, Japan; 2 Centre for Birth Cohort Studies, University of Yamanashi, 1110 Shimokato, Chuo, Yamanashi, Japan

**Keywords:** Unplanned pregnancy, Unintended pregnancy, Rural women, Prevalence, Risk factors, Diet, Smoking, Family planning, Contraceptive methods, Japan

## Abstract

**Background:**

Unplanned pregnancy is a public health issue with adverse consequences for maternal and neonatal health. In Japan, the prevalence of unplanned pregnancy was 46.2% in 2002. However, few studies have investigated this topic, and there is little recent data from Japan. We described and examined the prevalence and determinants of unplanned pregnancy among rural women in Japan from 2011 to 2016.

**Methods:**

We used cross-sectional data from a community-based cohort study (Project Koshu). Data were collected from 2011 to 2016 via a self-report questionnaire included in the Maternal and Child Health Handbook of Japan. Pregnancy intention was measured as a binary variable (planned or unplanned). Univariate and multivariate logistic regression analyses were performed to examine factors associated with unplanned pregnancy, with results reported as odds ratios (ORs) and 95% confidence intervals (CIs). We conducted sensitivity analyses with different definitions of pregnancy intention to assess the robustness of the results. The significance level was set at 5%.

**Results:**

Of the 932 participants (mean ± standard deviation age at baseline: 31.3 ± 5.2 years), 382 (41%) pregnancies were reported as unplanned. The multivariate analyses showed that maternal age (+ 1 year: OR = 0.94, 95% CI: 0.92–0.97, *p* <  0.001), ‘other’ family structure (OR = 2.76, 95% CI: 1.12–6.76, *p* = 0.03), three or more pregnancies (OR = 2.26, 95% CI: 1.66–3.08, *p* <  0.001), current smoking (OR = 2.60, 95% CI: 1.26–5.35, *p* = 0.01), balanced diet (OR = 0.62, 95% CI: 0.47–0.83, *p* <  0.001) and current depression (OR = 1.63, 95% CI: 1.24–2.16, *p* <  0.001) were strongly associated with unplanned pregnancy. These associations were consistent across definitions of pregnancy intention, supporting the robustness of our results.

**Conclusions:**

The prevalence of unplanned pregnancy in the study population was high (41%). Risk factors for unplanned pregnancy were age, number of pregnancies, smoking, having a balanced diet and current depression. These results suggest greater efforts are needed to enhance sex education for young people, improve access to family planning services and provide comprehensive health care for high-risk women to help reduce unplanned pregnancies.

## Background

In 2012, there were 84.9 million unplanned pregnancies worldwide, with half of these ending in abortion [1]. Unplanned pregnancy can negatively affect both mothers’ and infants’ health [2]. Recent evidence suggests that women with unplanned pregnancies may defer receiving prenatal care, face higher risk of preterm delivery and suffer more obstetric complications [3–5]. In addition, infants born following unplanned pregnancies are more likely to suffer from congenital deformities and are at higher risk of not receiving good prenatal care and not being breastfed [3, 6]. Their birth weight may also be lower than that of planned new-born babies [7].

‘Unplanned pregnancy’ and ‘unintended pregnancy’ are used interchangeably in the literature, and measures of this outcome vary across studies. For example, several studies have used an item that simply asked participants whether their pregnancy was planned or unplanned (intended or unintended). However, research in the United States has estimated the ratio of unintended pregnancies using a timing-based measure from the National Survey of Family Growth, and United Kingdom-based research has estimated the proportion of unplanned pregnancies using the London Measure of Unplanned Pregnancy [8–10]. Because of these differences, direct comparison of the results of published studies is challenging.

To date, there has been little agreement regarding what a ‘planned’ or ‘unplanned’ pregnancy is or how pregnancy intention should be measured and interpreted [11–14]. Previous research has established that operationalising pregnancy intention as a dichotomous variable may be insufficient to assess a woman’s intention regarding pregnancy [11]. Data from several sources have indicted an incongruence between a pregnant woman’s intention and feelings about the current pregnancy [15–18] for example, Trussell et al. highlighted that most women who experienced a pregnancy because of contraceptive failure felt happy or very happy about it [15]. Several lines of evidence have also suggested that women often profess happiness about pregnancies classified as unplanned [9, 19].

Nevertheless, numerous studies have found unplanned pregnancy to be associated with the same key factors: Specifically, these are maternal age [20–27], paternal age [23], low educational attainment [20–23, 25, 26], low socioeconomic status [22, 23, 25], pregnancy history [21–26], employment status [22, 25], smoking status [20, 21, 26, 28, 29], alcohol consumption [21, 26], drug use [20, 21, 26], healthy behaviours [28] and depression [20, 23]. In particular, data from the third National Survey of Sexual Attitudes and Lifestyles [20] in Britain showed that nearly half of unplanned pregnancies (45.2%) occurred among participants aged 16–19 years. Reproductive health items (i.e. having first intercourse at age 16 years or younger, non-use of contraception at first sex), low educational level, harmful health behaviours (i.e. drug use, current smoking) and current depression were also found to be positively correlated with unplanned pregnancy in that study. An analysis of the 2014 Ghana Demographic and Health Survey reported other factors associated with unplanned pregnancy, including the number of pregnancies, contraception use, marital status, socioeconomic status and occupational status [22].

Published data on unplanned pregnancy in Japan are scarce. A study in Yamagata, Japan, found the prevalence of unplanned pregnancy to be 46.2% and reported associations between unplanned pregnancy and maternal age, age at initiation of sexual intercourse, age at first marriage and spousal age difference [30]. However, these results were based on data from approximately 20 years ago and were obtained from participants in cervical and breast cancer screenings. Recently, another article reported that Japanese women still used effective contraceptive methods such as oral contraceptive pills (OCPs) relatively infrequently [31], but no single study has investigated how this affects the rate of unplanned pregnancies. Additionally, to our knowledge, no studies have specifically examined the effects of unplanned pregnancy on individuals and society in Japan.

Preventing unplanned pregnancies and improving maternal health care services are crucial for reducing maternal deaths, which is part of Sustainable Development Goal 3: ‘Reducing the global maternal mortality rate to less than 70 per 100,000 births’ by 2030 [32]. Drawing data from a community-based prospective cohort study, we investigated the prevalence of unplanned pregnancy and associated factors among women living in a rural area of Japan. The study aimed to contribute to the nascent body of research on unplanned pregnancy in Japan and to provide fresh insight into this major reproductive health issue to assist in the development of effective strategies for reducing the number of unplanned conceptions.

## Methods

The data used in this study were drawn from Project Koshu, which is an ongoing prospective cohort study for which recruitment began in 1988. Koshu City is a rural area in Yamanashi Prefecture, Japan, with a population of 32,188 people as of 1 October 2014, which represents about 3.8% of the total population of Yamanashi Prefecture (840,139). Expectant mothers were enrolled in Project Koshu at the Koshu City Office.

All participants were informed in writing of the aim of Project Koshu. They then provided their data from the Maternal and Child Health (MCH) Handbook of Japan, along with their signatures. Participation was voluntary. We analysed the data anonymously. This study was approved by the Ethics Review Board of the School of Medicine, University of Yamanashi (approval number: 1398) in accordance with the provisions of the Declaration of Helsinki and the ‘Guidelines Concerning Epidemiological Research’ of Japan. A detailed description of the project has been published elsewhere [33–35].

The Strengthening the Reporting of Observational Studies in Epidemiology guidelines [36] were used to ensure appropriate reporting of our study’s design, conduct and findings (Supplementary Material 1). In the present study, we analysed cross-sectional data on pregnant women participating in Project Koshu from 2011 to 2016. All variables were obtained through a self-report questionnaire included in the MCH Handbook. The MCH Handbook is a national programme in Japan that involves a 49-page booklet in which pregnancy, delivery, child development and health care information is recorded to inform all health care workers and other relevant service providers [37].

### Outcome and exposures

Pregnancy intention, which was the primary outcome of our research, was assessed with the question ‘Is this pregnancy planned?’ The response options were: (1) *yes*; (2) *no* and (3) *I cannot say either way*. We categorized this outcome as a dichotomous variable: If a participant answered *yes*, her pregnancy was considered planned; if she chose response (2) or (3), the pregnancy was considered unplanned.

Despite the lack of a generally accepted definition of pregnancy intention, there is no doubt about the detrimental effects of unplanned pregnancy on maternal and child health. Thus, to avoid inadvertently omitting any such vulnerable pregnancies, we kept respondents who said *I cannot say either way* in the analysis, classifying them as having unplanned pregnancies.

There were certainly drawbacks to this proposed definition. First, it would have been desirable to have further empirical justification for including the pregnancies of participants who felt unsure about whether the pregnancy was planned in the group of unplanned pregnancies, but the data required for this were not available in our study. Additionally, our use of a single item to assess pregnancy intention was potentially problematic. To address this potential problem, we used a statistical method to assess the robustness of our results, as described in the following subsection.

The independent variables, chosen on the basis of a review of the relevant literature, were maternal age, paternal age, family structure, number of pregnancies (defined as the number of previous pregnancies + 1), maternal employment status, smoking status, drinking status, having a balanced diet and currently being depressed. These covariates were obtained through the self-report questionnaire included in the MCH Handbook. The categorization of all covariates is shown in Table 1.

**Table 1:** Prevalence of unplanned pregnancy and baseline characteristics among 932 participants, 2011–2016

Mean ± standard deviation or number (%)	
Maternal age, years	31.3 ± 5.2
Paternal age, years	33.4 ± 6.3
**Pregnancy intention**	
Planned	550 (59.0)
Unplanned	382 (41.0)
**Family structure**	
Nuclear family	620 (66.5)
Extended family	286 (30.7)
Other	26 (2.8)
**Number of pregnancies**	
1–2	633 (67.9)
≥ 3	299 (32.1)
**Employment status**	
Employed	639 (68.6)
Unemployed	293 (31.4)
**Smoking status**	
Never smoked	647 (69.4)
Ex-smoker	244 (26.2)
Current smoker	41 (4.4)
**Drinking status**	
Never drank	479 (51.4)
Ex-drinker	412 (44.2)
Current drinker	41 (4.4)
**Balanced diet**	
No	431 (46.2)
Yes	501 (53.8)
**Currently depressed**	
No	519 (55.7)
Yes	413 (44.3)

### Statistical analysis

The associations between unplanned pregnancy and the above factors were first investigated with univariate logistic regression and then with multivariate logistic models fitted using a stepwise approach [38]. For each categorical variable except for having a balanced diet, we chose the normative group as the reference category (i.e. nuclear family, number of pregnancies ≤2, employed status, never smoked, never drank, not currently depressed). For balanced diet, we selected not having a balanced diet as the reference category because we wanted to compare women with a healthy lifestyle with those who did not have a healthy lifestyle to observe any differences in terms of planned/unplanned pregnancy status. We measured the impact of multicollinearity among the independent variables in our regression model using the variance inflation factor (VIF), with a cutoff of 5 (i.e. VIF > 5 was taken to indicate high multicollinearity) [39].

We also conducted sensitivity analyses to explore the uncertainty regarding using a single item to measure pregnancy intention. This method has been described as particularly useful in investigating “the robustness of an assessment by examining the extent to which results are affected by changes in methods, models, values of unmeasured variables, or assumptions” [40]. The findings in terms of the risk factors for unplanned pregnancy were expected to be consistent under different definitions of the outcome, which would indicate that our results were robust and that the original definition of unplanned pregnancy reflected the condition of interest well [41].

In the sensitivity analyses, we therefore introduced a second definition of the outcome by considering responses to an additional question, which asked about the participant’s feeling about the pregnancy (Question 2, five answer options; Supplementary Material 2). This additional variable was included on the basis of previous studies that have shown a lack of congruence between pregnancy intention and feeling about the pregnancy (i.e. women often feel happy about the pregnancy classified as unplanned) [9, 19]. Specifically, in this definition of an unplanned pregnancy, a pregnancy was considered planned if a participant reported that it was planned (i.e. answered *yes* when asked ‘Is this pregnancy planned?’) and felt happy about the pregnancy; otherwise, the pregnancy was considered unplanned (i.e. the participant reported that the pregnancy was unplanned or did not feel happy about the pregnancy).

The data are presented as crude and adjusted odds ratios (ORs) with 95% confidence intervals (CIs). A *p*-value of < 0.05 was considered statistically significant. All analyses were performed using R software, Version 3.6.0 (2019-04-26).

## Results

Table 1 shows the overall characteristics of the 932 pregnant women who completed the questionnaire when they registered their pregnancies at the City Office in Koshu City, Yamanashi Prefecture, Japan, from 2011 to 2016. The mean age of participating women was 31.3 years and that of their partners was 33.4 years. Few women (2.8%) reported that their family structure was ‘other’ (i.e. something other than a nuclear or extended family). Approximately two-thirds of the participants had been pregnant once or twice (including the current pregnancy), and 68.6% were employed. The majority of the respondents reported healthy lifestyle behaviours, including never smoking (69.4%), never consuming alcohol (51.4%) and having a balanced diet (53.8%). However, 413 women (44.3%) reported that they were currently experiencing depressed mood.

In response to the question, ‘Is this pregnancy planned?’, 59% of the participants chose *yes*. This meant that 41% of the pregnancies were unplanned (Table 1). In addition, most of the women (85%) felt happy about being pregnant; only 1.2% selected *do not feel anything*, and 35 participants (3.8%) reported their feelings as *other* (Supplementary Material 2).

The associations between pregnancy intention and the independent variables were tested using logistic regression analyses. We found no evidence of multicollinearity among the predictors: All VIF values were around 1, except for the values for maternal age and paternal age, which were 2.16 and 2.04, respectively (Supplementary Material 3). Table 2 shows that, in the univariate analyses, there were significant associations between unplanned pregnancy and younger maternal age, paternal age, ‘other’ family structure, three or more pregnancies and currently smoking. Having a balanced diet and currently being depressed were also independent predictors of unplanned pregnancy. We did not find any statistically significant associations between pregnancy intention and women’s employment or drinking status. The multivariable model fitted using a stepwise approach showed that younger maternal age, ‘other’ family structure, three or more pregnancies, currently smoking, having a balanced diet and currently being depressed were factors associated with unplanned pregnancy. These results are summarised in Table 2.

**Table 2:** Risk factors for unplanned pregnancy among Japanese mothers

	Crude analysis OR (95% CI)	*p*-value	Multivariable analysis OR (95% CI)	*p*-value
**Maternal age**				
+ 1 year	0.95 (0.93–0.97)	< 0.001	0.94 (0.92–0.97)	< 0.001
**Paternal age**				
+ 1 year	0.96 (0.94–0.98)	< 0.001	–	–
**Family structure**				
Nuclear family	Ref	–	Ref	–
Extended family	1.11 (0.84–1.48)	0.46	1.01 (0.75–1.37)	0.93
Other	3.47 (1.48–8.10)	0.004	2.76 (1.12–6.76)	0.027
**Number of pregnancies**				
1–2	Ref	–	Ref	–
≥ 3	1.71 (1.29–2.25)	< 0.001	2.26 (1.66–3.08)	< 0.001
**Employment status**				
Employed	Ref	–	–	–
Unemployed	1.08 (0.82–1.43)	0.58		
**Smoking status**				
Never smoked	Ref	–	Ref	–
Ex-smoker	1.13 (0.84–1.53)	0.41	1.06 (0.77–1.45)	0.72
Current smoker	3.81 (1.91–7.61)	< 0.001	2.60 (1.26–5.35)	0.01
**Drinking status**				
Never drank	Ref	–	–	–
Ex-drinker	0.82 (0.62–1.07)	0.14		
Current drinker	1.14 (0.6–2.17)	0.68		
**Balanced diet**				
No	Ref	–	Ref	–
Yes	0.55 (0.42–0.72)	< 0.001	0.62 (0.47–0.83)	< 0.001
**Currently depressed**				
No	Ref	–	Ref	–
Yes	1.74 (1.34–2.26)	< 0.001	1.63 (1.24–2.16)	< 0.001

Multivariable model fitted using stepwise regression. *OR *odds ratio; *CI* confidence interval; *Ref* reference

Table 3 shows the results of the multivariate logistic regression analyses with two different definitions of unplanned pregnancy. Notably, the main analysis and the sensitivity analysis revealed almost the same risk factors for unplanned pregnancy. Therefore, our results can be considered robust.

**Table 3:** Multivariate analysis results for two definitions of unplanned pregnancy

	Main analysis^**(a)**^	***p***-value	Sensitivity analysis^**(b)**^	***p***-value
**Maternal age**				
+ 1 year	0.94 (0.92–0.97)	< 0.001	0.95 (0.93–0.98)	< 0.001
**Family structure**				
Nuclear family	Ref	–	Ref	–
Extended family	1.01 (0.75–1.37)	0.93	1.03 (0.77–1.38)	0.86
Other	2.76 (1.12–6.76)	0.027	3.03 (1.21–7.60)	0.018
**Number of pregnancies**				
1–2	Ref	–	Ref	–
≥ 3	2.26 (1.66–3.08)	< 0.001	2.19 (1.62–2.97)	< 0.001
**Smoking status**				
Never smoked	Ref	–	Ref	–
Ex-smoker	1.06 (0.77–1.45)	0.72	1.02 (0.75–1.39)	0.90
Current smoker	2.60 (1.26–5.35)	0.01	2.17 (1.06–4.45)	0.035
**Balanced diet**				
No	Ref	–	Ref	–
Yes	0.62 (0.47–0.83)	< 0.001	0.67 (0.51–0.88)	0.004
**Currently depressed**				
No	Ref	–	Ref	–
Yes	1.63 (1.24–2.16)	< 0.001	1.73 (1.32–2.28)	< 0.001

Pregnancy intention is measured using a single item: ‘Is this pregnancy planned?’ in (^a^) and by a combination of that item and a variable gauging women’s feelings about the pregnancy in (^b^). Details of the definition are described in the Methods section.

The data are presented as odds ratios with 95% confidence intervals. *Ref* reference

## Discussion

Reducing the number of unplanned pregnancies is a critical goal of family planning programmes. To achieve this goal, health policy-makers need to be provided with information about the prevalence of and factors associated with unplanned pregnancy. To obtain that information, we conducted a secondary analysis of baseline data from Project Koshu, a cohort study conducted in a rural area of Japan from 1998 to 2016. Of the 932 women who completed the Project Koshu questionnaire from 2011 to 2016, 41% reported that their pregnancies were unplanned. We found that young women, women who experienced three or more pregnancies (including the current pregnancy), women whose family structure was neither nuclear nor extended, current smokers, women with an unbalanced diet and women who were currently depressed had a higher odds of experiencing unplanned pregnancy.

### Interpretation in the context of previous studies

Our findings for the prevalence of unplanned pregnancy were similar to worldwide findings (40%) and higher than those for Asian countries (38%) reported in a 2012 study [1]. When compared with a study conducted in 2002 in Japan [30], the present study showed that the prevalence of unplanned pregnancy had decreased (from 46.2 to 41%), although it remained high. This relatively high prevalence of unplanned pregnancy may reflect an unmet need for contraception in our study population, especially because the rate of OCP use was low (3.0%) in 2014, reflecting little change since the OCP was legalised in 1999 [31].

These results should be interpreted with caution because we kept the participants who did not provide a definite answer of *yes* or *no* when asked ‘Is this pregnancy planned?’ and considered their pregnancies to be unplanned. While there is no unambiguous definition of pregnancy intention, the correspondence between the prevalence of unplanned pregnancy and current trends in contraceptive use in Japan discussed above appears to support our definition. Moreover, from a practical perspective, this definition would enable expectant mothers with ambiguous pregnancy intentions to benefit from appropriate support and counselling throughout pregnancy. Our findings thus have the potential to inform policy in ways that benefit not only the present study population, but also other women in Japan and in other contexts where information on pregnancy, delivery and child health is similarly recorded.

In this study, maternal and paternal age were associated with unplanned pregnancy, which was consistent with previous studies conducted in Japan and elsewhere [20, 30]. Our findings showed that younger women were more likely to report an unplanned pregnancy (adjusted OR = 0.94, 95% CI: 0.92–0.97; Table 2). This differed from the findings of a previous study in Bangladesh [27], which showed that, for each additional year of maternal age, the odds of unplanned pregnancy increased by 15% (unadjusted OR = 1.15, 95% CI: 1.01–2.12), suggesting that older women appeared to have already had the ideal number of children and did not desire further pregnancies. Moreover, older women tended to use contraception less frequently [42], whereas younger women lacked knowledge about safe sex and effective contraceptives [43–45]. The results from our study and other studies suggest that women at the two poles of reproductive age need to be made aware of the need for appropriate contraceptive methods.

The present study also found that unplanned pregnancy was significantly associated with the women’s family structure and number of pregnancies. As shown in Table 2, women whose family structure was categorised as ‘other’ were 2.76 times more likely to have an unplanned pregnancy (adjusted OR = 2.76, 95% CI: 1.12–6.76). Our findings also expand on prior work reporting that the initiation of sexual intercourse occurred earlier among adolescents from single-parent families [46] and that teenagers from nuclear families had a lower level of sexual experience compared with their counterparts [47]. In addition, women who reported three or more pregnancies had higher odds of experiencing unplanned pregnancy than did those who had one or two pregnancies (adjusted OR = 2.26, 95% CI: 1.66–3.08). These results were consistent with those from previous research [48–50]. For example, a study conducted in Ethiopia revealed that a higher number of pregnancies was associated with a greater chance of a woman experiencing an unplanned pregnancy (adjusted OR = 3.16, 95% CI: 1.37–7.30) [49]. A possible explanation for this is that, because high-parity women already have their ideal number of children, they may be more likely to report later pregnancies as unplanned [50].

Another important finding in our study involved the relationships between maternal behaviours and pregnancy intention. A study conducted in the United States [51] reported that women with a folic acid deficiency had higher odds of unplanned pregnancy, compared with those with adequate folic acid levels (adjusted OR = 2.17, 95% CI: 1.78–2.64), which was in line with our finding of lower odds of unplanned pregnancy among women with a balanced diet (adjusted OR = 0.62, 95% CI: 0.47–0.83). A note of caution is in order here because dietary quality was assessed using a single-item self-rated question; however, the strong relationship observed suggests that women who adopt and maintain healthy behaviours may be more likely to plan when to become pregnant. Supporting evidence from previous observations [20, 51], our results also indicated that women who were current smokers had higher odds of unplanned pregnancy than did those who had never smoked (adjusted OR = 2.60, 95% CI: 1.26–5.35).

Several reports have shown an association between depression and pregnancy intention [20, 52, 53]. This relationship was confirmed by our finding that women who were currently depressed had 63% higher odds of unplanned pregnancy than did women who were not currently depressed (adjusted OR = 1.63, 95% CI: 1.24–2.16). This result may be explained by people with higher levels of depressive symptoms having a tendency to indulge in more frequent sexual intercourse without considering contraceptive use [54–56]. However, further work is required to establish causality. Women who are depressed may need more attention because they may be more susceptible to unplanned pregnancy, and women with an unplanned pregnancy should be screened for depression early in prenatal care.

Unlike some research conducted in this area, we did not observe any statistically significant correlations between unplanned pregnancy and women’s employment or drinking status. This difference may be attributable to variations in sample characteristics, as noted by Hall et al. [23].

### Practical implications

Using effective contraception is a key factor in preventing unplanned pregnancies. About 50% of unplanned pregnancies in the United States and 65% in France have been shown to result from contraceptive failure [57, 58]. Prior studies have noted that increasing access to no-cost contraception may help women to avoid unplanned pregnancies [59, 60]. Among the methods of contraception, long-acting reversible contraceptives, including intrauterine devices and contraceptive implants, are recommended because of their higher efficacy and continuation rates compared with other reversible methods [61, 62].

A Japanese study on current contraceptive use trends revealed that condoms were the most popular method (83.4%), whereas the OCP was the least used method (3.0%) [31]. The most common causes of this low rate of OCP use have been reported as worrying side effects, the cost of the method and the burden of obtaining a doctor’s prescription [63]. OCPs and other commonly available methods (e.g. condoms, spermicides and intrauterine devices) are not covered by national health insurance in Japan, and most OCP prescriptions are obtained from gynaecologists. This suggests that crucial strategies to help reduce the number of unplanned pregnancies include more extensive training programmes for health care professionals, increasing knowledge about contraceptive use among women of reproductive age, and increasing contraceptive access and choice (especially for the more effective contraceptive methods such as long-acting reversible contraceptives). Finally, our findings regarding the prevalence of unplanned pregnancy, combined with the previous report that this prevalence was relatively high in 2002 [30], underscores the need for future research exploring the negative impact of unplanned pregnancy on individuals and society in Japan to improve reproductive health and public health policy.

Several previous studies have investigated the associations of maternal smoking behaviour with low birth weight and childhood development [34, 35]. The present study found that current smokers were more likely to experience unplanned pregnancy. A nationwide longitudinal study in Japan conducted from 2005 to 2014 found a positive correlation between smoking cessation and alcohol cessation (unadjusted OR = 1.86, 95% CI 1.08–3.20) and a negative association between smoking cessation and stopping routine medical check-ups (unadjusted OR = 0.31, 95% CI 0.18–0.53) [64]. It is important that health care providers discuss smoking cessation with pregnant smokers and provide them with appropriate interventions such as behavioural counselling, which is the most recommended approach to smoking cessation for pregnant women [65].

### Strengths and limitations

The strengths of this study are that the assessment of pregnancy intention was not influenced by pregnancy outcome and that the risk of recall bias was reduced because the data were collected when participants were registering their pregnancy at the City Office. However, there are also several limitations.

First, other scholars have expressed concerns about how planned and unplanned pregnancies are defined (e.g. because a woman’s responses about a pregnancy she has classified as unplanned may be contradictory or ambivalent), as well as about inconsistencies between a woman’s intention and her feelings about the pregnancy. Therefore, using a single item to measure pregnancy intention may be a limitation of our study. However, we found that the results for factors associated with unplanned pregnancy did not change when we had changed the definition of the outcome. Moreover, our study’s findings on the prevalence of unplanned pregnancy appear to well reflect current trends in contraceptive use in Japan, and the identified factors that are associated with unplanned pregnancy are consistent with the factors found to be associated with unplanned pregnancy in previous studies in other contexts. These support the robustness of our findings, encourage the definition of unplanned pregnancy used in the main analysis and build on previous literature.

Second, because of the cross-sectional study design, we could not determine causal links between pregnancy intention and the identified risk factors. Third, the findings cannot be generalised to the whole of Japan or to other settings because the study was set in one rural area in Japan. Further nationwide studies that consider this issue are needed to develop a comprehensive picture of unplanned pregnancy and its associated factors.

## Conclusions

This study showed that the prevalence of unplanned pregnancy in the study setting was relatively high (41%), demonstrating the need for more attention to this issue. Family planning programmes should be widely and easily accessible to women of reproductive age, especially those at high risk for unplanned pregnancy. In particular, our findings suggest that there is a need for better sex education and increased knowledge about contraceptive methods for young people. In addition, multigravidas and women without family support may need individualised instruction and counselling to increase the effectiveness and consistency of use of contraceptive methods. Finally, mental health care and interventions for health behaviour modification should be implemented from preconception throughout pregnancy to prevent unplanned pregnancy and related negative outcomes.

## Supplementary information

**Additional file 1 **STROBE Statement—Checklist of items that should be included in reports of *cross-sectional studies* [36].

**Additional file 2.** Questions and distribution of answers about pregnancy intention.

**Additional file 3.** Variance inflation factor (VIF) results.

## Data Availability

The data sets analysed in the current study are available from author Zentaro Yamagata (zenymgt@yamanashi.ac.jp) upon request.

## Abbreviations

CI: Confidence interval
MCH Handbook: Maternal and Child Health Handbook
OCP: Oral contraceptive pill
OR: Odds ratio
VIF: Variance inflation factor

## References

[1] Sedgh G, Singh S, Hussain R (2014). Intended and unintended pregnancies worldwide in 2012 and recent trends. Stud Fam Plan.

[2] Brown SS, Eisenberg L (1995). The best intentions.

[3] Korenman S, Kaestner R, Joyce T (2002). Consequences for infants of parental disagreement in pregnancy intention. Perspect Sex Reprod Health.

[4] Shah PS, Balkhair T, Ohlsson A, Beyene J, Scott F, Frick C (2011). Intention to become pregnant and low birth weight and preterm birth: a systematic review. Matern Child Health J.

[5] Mohllajee AP, Curtis KM, Morrow B, Marchbanks PA (2007). Pregnancy intention and its relationship to birth and maternal outcomes. Obstet Gynecol.

[6] Blomberg S (1980). Influence of maternal distress during pregnancy on fetal malformations. Acta Psychiatr Scand.

[7] Kost K, Lindberg L (2015). Pregnancy intentions, maternal behaviors, and infant health: investigating relationships with new measures and propensity score analysis. Demography..

[8] ESHRE Capri Workshop Group (2018). Why after 50 years of effective contraception do we still have unintended pregnancy? A European perspective. Hum Reprod.

[9] Chandra A, Martinez GM, Mosher WD, Abma JC, Jones J (2005). Fertility, family planning, and reproductive health of U.S. women: data from the 2002 National Survey of Family Growth. Vital Health Stat.

[10] Barrett G (2004). Conceptualisation, development, and evaluation of a measure of unplanned pregnancy. J Epidemiol Community Heal.

[11] Aiken ARA, Dillaway C, Mevs-Korff N (2015). A blessing I can’t afford: factors underlying the paradox of happiness about unintended pregnancy. Soc Sci Med.

[12] Barrett G, Wellings K (2002). What is a ‘planned’ pregnancy? Empirical data from a British study. Soc Sci Med.

[13] Santelli J, Rochat R, Hatfield-Timajchy K, Gilbert BC, Curtis K, Cabral R, et al. The measurement and meaning of unintended pregnancy. Perspect Sex Reprod Health. 35:94–101. 10.1363/3509403 12729139

[14] Aiken ARA, Westhoff CL, Trussell J, Castaño PM (2016). Comparison of a timing-based measure of unintended pregnancy and the London measure of unplanned pregnancy. Perspect Sex Reprod Health.

[15] Trussell J, Vaughan B, Stanford J (1999). Are all contraceptive failures unintended pregnancies? Evidence from the 1995 National Survey of family growth. Fam Plan Perspect.

[16] Rosenzweig MR, Wolpin KI (1993). Maternal expectations and ex post rationalizations: the usefulness of survey information on the Wantedness of children. J Hum Resour.

[17] Aiken ARA, Potter JE (2013). Are Latina women ambivalent about pregnancies they are trying to prevent? Evidence from the border contraceptive access study. Perspect Sex Reprod Health.

[18] Crosby RA, DiClemente RJ, Wingood GM, Davies SL, Harrington K (2002). Adolescents’ ambivalence about becoming pregnant predicts infrequent contraceptive use: a prospective analysis of nonpregnant African American females. Am J Obstet Gynecol.

[19] Hartnett CS (2012). Are Hispanic women happier about unintended births?. Popul Res Policy Rev.

[20] Wellings K, Jones KG, Mercer CH, Tanton C, Clifton S, Datta J (2013). The prevalence of unplanned pregnancy and associated factors in Britain: findings from the third National Survey of sexual attitudes and lifestyles (Natsal-3). Lancet..

[21] Oulman E, Kim THM, Yunis K, Tamim H (2015). Prevalence and predictors of unintended pregnancy among women: an analysis of the Canadian maternity experiences survey. BMC Pregnancy Childbirth.

[22] Ameyaw EK (2018). Prevalence and correlates of unintended pregnancy in Ghana: analysis of 2014 Ghana demographic and health survey. Matern Heal Neonatol Perinatol.

[23] Hall JA, Barrett G, Phiri T, Copas A, Malata A, Stephenson J (2016). Prevalence and determinants of unintended pregnancy in Mchinji district, Malawi; using a conceptual hierarchy to inform analysis. PLoS One.

[24] Getu Melese K, Gebrie MH, Berta Badi M, Fekadu MW (2016). Unintended pregnancy in Ethiopia: community based cross-sectional study. Obstet Gynecol Int.

[25] Iseyemi A, Zhao Q, McNicholas C, Peipert JF (2017). Socioeconomic status as a risk factor for unintended pregnancy in the contraceptive CHOICE project. Obstet Gynecol.

[26] Than LC, Honein MA, Watkins ML, Yoon PW, Daniel KL, Correa A (2005). Intent to become pregnant as a predictor of exposures during pregnancy: is there a relation?. J Reprod Med.

[27] Rahman M (2012). Women’s autonomy and unintended pregnancy among currently pregnant women in Bangladesh. Matern Child Health J.

[28] Hellerstedt WL, Pirie PL, Lando HA, Curry SJ, McBride CM, Grothaus LC (1998). Differences in preconceptional and prenatal behaviors in women with intended and unintended pregnancies. Am J Public Health.

[29] Flower A, Shawe J, Stephenson J, Doyle P (2013). Pregnancy planning, smoking behaviour during pregnancy, and neonatal outcome: UK millennium cohort study. BMC Pregnancy Childbirth..

[30] Goto A, Yasumura S, Reich MR, Fukao A (2002). Factors associated with unintended pregnancy in Yamagata, Japan. Soc Sci Med.

[31] Yoshida H, Sakamoto H, Leslie A, Takahashi O, Tsuboi S, Kitamura K (2016). Contraception in Japan: current trends. Contraception..

[32] United Nations. Sustainable Development Goal 3: Ensure healthy lives and promote well-being for all at all ages. https://sustainabledevelopment.un.org/sdg3. Accessed 19 May 2020.

[33] Suzuki K, Kondo N, Sato M, Tanaka T, Ando D, Yamagata Z (2011). Gender differences in the association between maternal smoking during pregnancy and childhood growth trajectories: multilevel analysis. Int J Obes.

[34] Mizutani T, Suzuki K, Kondo N, Yamagata Z (2007). Association of maternal lifestyles including smoking during pregnancy with childhood obesity. Obesity..

[35] Suzuki K, Tanaka T, Kondo N, Minai J, Sato M, Yamagata Z (2008). Is maternal smoking during early pregnancy a risk factor for all low birth weight infants?. J Epidemiol.

[36] von Elm E, Altman DG, Egger M, Pocock SJ, Gøtzsche PC, Vandenbroucke JP (2007). The strengthening the reporting of observational studies in epidemiology (STROBE) statement: guidelines for reporting observational studies. Ann Intern Med.

[37] Takeuchi J, Sakagami Y, Perez RC (2016). The Mother and Child Health Handbook in Japan as a Health Promotion Tool. Glob Pediatr Heal.

[38] Hosmer DW, Lemeshow S, Quigley S (2000). Model-building strategies and methods for logistic regression. Applied logistic regression.

[39] Craney TA, Surles JG (2002). Model-dependent variance inflation factor cutoff values. Qual Eng.

[40] Schneeweiss S (2006). Sensitivity analysis and external adjustment for unmeasured confounders in epidemiologic database studies of therapeutics. Pharmacoepidemiol Drug Saf.

[41] Thabane L, Mbuagbaw L, Zhang S, Samaan Z, Marcucci M, Ye C (2013). A tutorial on sensitivity analyses in clinical trials: the what, why, when and how. BMC Med Res Methodol.

[42] Wu J, Meldrum S, Dozier A, Stanwood N, Fiscella K (2008). Contraceptive nonuse among US women at risk for unplanned pregnancy. Contraception..

[43] Magadi M (2006). Poor pregnancy outcomes among adolescents in South Nyanza region of Kenya. Afr J Reprod Health.

[44] Forrest JD (1994). Epidemiology of unintended pregancy and contraceptive use. Am J Obstet Gynecol.

[45] Izugbaraa CO, Ochako R, Izugbara C (2011). Gender scripts and unwanted pregnancy among urban Kenyan women. Cult Health Sex.

[46] Miller BC, Benson B, Galbraith KA (2001). Family relationships and adolescent pregnancy risk: a research synthesis. Dev Rev.

[47] Young EW, Jensen LC, Olsen JA, Cundick BP (1991). The effects of family structure on the sexual behavior of adolescents. Adolescence..

[48] Vázquez-Nava F, Vázquez-Rodriguez CF, Saldívar-González AH, Vázquez-Rodríguez EM, Córdova-Fernández JA, Felizardo-Ávalos J (2014). Unplanned pregnancy in adolescents: association with family structure, employed mother, and female friends with health-risk habits and behaviors. J Urban Heal.

[49] Hamdela B, Mariam GA, Tilahun T (2012). Unwanted pregnancy and associated factors among pregnant married women in Hosanna town, Southern Ethiopia. PLoS One.

[50] Mohammed F, Musa A, Amano A (2016). Prevalence and determinants of unintended pregnancy among pregnant woman attending ANC at Gelemso general hospital, Oromiya region, East Ethiopia: A facility based cross-sectional study. BMC Womens Health.

[51] Cheng D, Schwarz EB, Douglas E, Horon I (2009). Unintended pregnancy and associated maternal preconception, prenatal and postpartum behaviors. Contraception..

[52] James-Hawkins L, Denardo D, Blalock C, Mollborn S (2014). Do depressive symptoms in male and female adolescents predict unintended births in emerging adulthood?. Matern Child Health J.

[53] Baba S, Kimura T, Ikehara S, Honjo K, Eshak ES, Sato T, et al. Impact of intention and feeling toward being pregnant on postpartum depression: the Japan environment and Children’s study (JECS). Arch Womens Ment Health. 2018. 10.1007/s00737-018-0938-7. 10.1007/s00737-018-0938-7 PMC6987065 30591966

[54] Kowaleski-Jones L, Mott FL (1998). Sex, contraception and childbearing among high-risk youth: do different factors influence males and females?. Fam Plann Perspect.

[55] Kirby D (2002). Antecedents of adolescent initiation of sex, contraceptive use, and pregnancy. Am J Health Behav.

[56] Chewning B, Koningsveld R (1998). Predicting adolescents’ initiation of intercourse and contraceptive use. J Appl Soc Psychol.

[57] Black KI, Gupta S, Rassi A, Kubba A (2010). Why do women experience untimed pregnancies? A review of contraceptive failure rates. Best Pract Res Clin Obstet Gynaecol.

[58] Kost K, Singh S, Vaughan B, Trussell J, Bankole A (2008). Estimates of contraceptive failure from the 2002 National Survey of family growth. Contraception..

[59] Peipert JF, Madden T, Allsworth JE, Secura GM (2012). Preventing unintended pregnancies by providing no-cost contraception. Obstet Gynecol.

[60] Rose SB, Cooper AJ, Baker NK, Lawton B (2011). Attitudes toward long-acting reversible contraception among Young women seeking abortion. J Women’s Heal.

[61] Blumenthal PD, Voedisch A, Gemzell-Danielsson K (2011). Strategies to prevent unintended pregnancy: increasing use of long-acting reversible contraception. Hum Reprod Update.

[62] Peipert JF, Zhao Q, Allsworth JE, Petrosky E, Madden T, Eisenberg D (2011). Continuation and satisfaction of reversible contraception. Obstet Gynecol.

[63] Matsumoto Y, Yamabe S, Sugishima T, Geronazzo D (2011). Perception of oral contraceptives among women of reproductive age in Japan: a comparison with the USA and France. J Obstet Gynaecol Res.

[64] Oshio T (2018). Association between successful smoking cessation and changes in marital and job status and health behaviours: evidence from a 10-wave nationwide survey in Japan. BMC Public Health.

[65] Siu AL (2015). Behavioral and pharmacotherapy interventions for tobacco smoking cessation in adults, including pregnant women: U.S. preventive services task force recommendation statement. Ann Intern Med.

