# Supplementary material for: Prevalence of and factors associated with unplanned pregnancy among women in Koshu, Japan: cross-sectional evidence from Project Koshu, 2011–2016
Source: BMC Pregnancy Childbirth. 2020 Jul 9;20:397. doi: 10.1186/s12884-020-03088-3 (PMC7346350; doi:10.1186/s12884-020-03088-3)
Supplement: Supplementary file 2 — Additional file 2. Questions and distribution of answers about pregnancy intention. [file 12884_2020_3088_MOESM2_ESM.docx]

**Supplementary Material 2.** Questions and distribution of answers about pregnancy intention

|  | Number (%) |
| --- | --- |
| Question 1: ‘Is this pregnancy planned’ |  |
| Yes  No  I cannot say either way | 550 (59.0)  176 (18.9)  206 (22.1) |
| Question 2: Feeling about this pregnancy |  |
| Happy  Embarrassed  Do not feel anything  Troubled  Other | 792 (85.0)  40 (4.3)  11 (1.2)  54 (5.8)  35 (3.8) |
