# Supplementary material for: Prevalence of and factors associated with unplanned pregnancy among women in Koshu, Japan: cross-sectional evidence from Project Koshu, 2011–2016
Source: BMC Pregnancy Childbirth. 2020 Jul 9;20:397. doi: 10.1186/s12884-020-03088-3 (PMC7346350; doi:10.1186/s12884-020-03088-3)
Supplement: Supplementary file 3 — Additional file 3. Variance inflation factor (VIF) results. [file 12884_2020_3088_MOESM3_ESM.docx]

**Supplementary Material 3.** Variance inflation factor (VIF) results

| **Variable** | **VIF** |
| --- | --- |
| Maternal age | 2.16 |
| Paternal age | 2.04 |
| Family structure | 1.16 |
| Number of pregnancies | 1.04 |
| Employment status | 1.02 |
| Smoking status | 1.09 |
| Drinking status | 1.06 |
| Balanced diet | 1.05 |
| Currently depressed | 1.03 |
